# Characteristics of DNA polymerase I from an extreme thermophile, *Thermus scotoductus* strain K1

**DOI:** 10.1002/mbo3.1149

**Published:** 2021-01-07

**Authors:** Ani Saghatelyan, Hovik Panosyan, Armen Trchounian, Nils‐Kåre Birkeland

**Affiliations:** ^1^ Department of Biochemistry, Microbiology and Biotechnology Yerevan State University Yerevan Armenia; ^2^ Department of Biological Sciences University of Bergen Bergen Norway

**Keywords:** DNA polymerase, fidelity, heterologous expression, PCR, thermostability, *Thermus*

## Abstract

Several native and engineered heat‐stable DNA polymerases from a variety of sources are used as powerful tools in different molecular techniques, including polymerase chain reaction, medical diagnostics, DNA sequencing, biological diversity assessments, and in vitro mutagenesis. The DNA polymerase from the extreme thermophile, *Thermus scotoductus* strain K1, (*Ts*K1) was expressed in *Escherichia coli*, purified, and characterized. This enzyme belongs to a distinct phylogenetic clade, different from the commonly used DNA polymerase I enzymes, including those from *Thermus* *aquaticus* and *Thermus* *thermophilus*. The enzyme demonstrated an optimal temperature and pH value of 72–74°C and 9.0, respectively, and could efficiently amplify 2.5 kb DNA products. *Ts*K1 DNA polymerase did not require additional K^+^ ions but it did need Mg^2+^ at 3–5 mM for optimal activity. It was stable for at least 1 h at 80°C, and its half‐life at 88 and 95°C was 30 and 15 min, respectively. Analysis of the mutation frequency in the amplified products demonstrated that the base insertion fidelity for this enzyme was significantly better than that of *Taq* DNA polymerase. These results suggest that *Ts*K1 DNA polymerase could be useful in various molecular applications, including high‐temperature DNA polymerization.

## Graphical abstract

The DNA polymerase from the extreme thermophile *Thermus scotoductus* strain K1 (*Ts*K1) was expressed in *Escherichia coli*, purified, and characterized. The enzyme demonstrated an optimal temperature and pH value of 72–74°C and 9.0, respectively, and could efficiently amplify 2.5 kb DNA products. *Ts*K1 DNA polymerase did not require additional K^+^ ions but it did need Mg^2+^ at 3–5 mM for optimal activity. The base insertion fidelity for this enzyme was significantly better than that of *Taq* DNA polymerase.

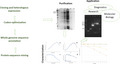

## 1 INTRODUCTION

DNA polymerase is an omnipresent enzyme that synthesizes complementary DNA strands from an existing template in living cells. DNA polymerases are widely used for a variety of molecular techniques that rely on DNA manipulation, including polymerase chain reaction (PCR), molecular cloning, sequencing, DNA labeling, and mutagenesis among others (Gibbs et al., 2009; Killelea et al., 2009; Coulther et al., 2019). Several thermostable DNA polymerases have been isolated and studied in prokaryotes (Cho et al., 2012; Kim et al., 2007; Lee et al., 2009; Terpe, 2013; Zhang et al., 2015). These enzymes are grouped into eight families: A, B, C, D, X, Y, RT, and AEP based on their amino acid sequences (Killelea et al., 2009; Coulther et al., 2019). Most thermostable DNA polymerases primarily used in PCR procedures belong to the A‐ and B‐family polymerases from thermophilic bacteria and archaea, respectively. Each thermostable DNA polymerase has its own set of unique characteristics, including thermostability, extension rate, fidelity, processivity, specificity, resistance to contaminants and inhibitors, modified nucleotide selection, ability to bypass damage, nuclease activity, and strand displacement activity. The distinctive properties of each DNA polymerase can be used to create unique reagents (Aschenbrenner & Marx, 2017; Coulther et al., 2019; Reha‐Krantz et al., 2014). This means that the search for novel DNA polymerases has been a major focus for the last couple of decades. A‐type polymerases from the genus *Thermus* are the most frequently used in molecular biology and include the commonly used *Taq* DNA polymerase from *T*.* aquaticus*. Several polymerases with similarities to *Taq* have been mined from other *Thermus* species, including *Tfi* from *T*.* filiformis*, *Tfl* from *T*.* flavus*, *Tbr* from *T*.* brockianus*, *Tca* from *T*.* caldophilus*, and *Tth* from *T*.* thermophilus*. Slight amino acid sequence differences between polymerase enzymes can result in dramatic changes to their biochemical characteristics, suggesting that it is possible to mine for novel polymerases with improved functionality (Gibbs et al., 2009). Other A‐type polymerases have been isolated from *Thermotoga* spp., including *Tma* polymerase from *T*.* maritima* and *Tne* from *T*.* neapolitana* (Spibida et al., 2017). Despite the number of available enzymes, the growing applications of molecular biology mean that there is still a demand for novel enzymes, and this is where the field of applied science might facilitate continued improvements.

Here, the expression, purification, and characterization of a recombinant DNA polymerase I from *Thermus scotoductus* strain K1 (*Ts*K1 DNA polymerase) originating from a geothermal spring in Karvachar, Nagorno‐Karabakh (Saghatelyan et al., 2015) is described.

## 2 MATERIALS AND METHODS

### 2.1 Source of enzyme

The enzyme was from *T*.* scotoductus* strain K1, which was isolated from a geothermal spring located in Karvachar, Nagorno‐Karabakh. The draft genome sequence of *T*.* scotoductus* K1 was deposited in the DBJ/EMBL/GenBank database under the RefSeq assembly accession no. GCF_001294665.1 (Saghatelyan et al., 2015). A phylogenetic tree depicting the evolutionary distance between *Ts*K1 DNA polymerase (Accession no. MW080815) and other *Thermus* spp. polymerases was constructed based on the JTT matrix model using the maximum likelihood method (Jones et al., 1992) in MEGA X software (Kumar et al., 2018). The *polI* codons were optimized (GenScript) to maximize expression in *E*.* coli* while maintaining the original amino acid sequence. The codon‐optimized gene was then synthesized by GenScript (https://www.genscript.com/).

### 2.2 Cloning

A pUC57‐Mini plasmid harboring the codon‐optimized *polI* sequence, 2512 bp, was used to facilitate the downstream cloning experiments completed using the FX (fragment exchange) system (Geertsma & Dutzler, 2011). The expression vector p7xC3H (6999 bp) (Addgene, LGC Standards), which contained a T7 promoter, a C‐terminal 3C protease cleavage site, and a C‐terminal 10× His tag, was used as the expression vector. Expression constructs were identified and maintained using kanamycin resistance conferred by the vector.

### 2.3 Protein expression and purification

1 L of 2×YT (yeast extract and tryptone) broth supplemented with 50 μg/ml kanamycin was used to culture *E*.* coli* BL21 (DE3) harboring the C‐10 recombinant expression plasmid at 37°C until an OD_600_ of 0.4 was obtained. Expression was induced by adding isopropyl β‐D‐1‐thiogalactopyranoside to 0.4 mM and further cultivation at 20 °C for another 16 h (at 200 rpm). Cell harvesting was performed by centrifugation followed by resuspension in buffer R (20 mM NaCl, 50 mM Tris‐HCl pH 7.5, 8% glycerol). The cells were then disrupted by lysozyme and freezing‐thawing, which was followed by sonication. The lysate was then heated at 70 °C for 20 min to precipitate most of the heat‐labile host proteins, and cell wall and insoluble debris were removed by further centrifugation at 7482 *g*, 4°C for 30 min. The supernatant was filtered using Whatman filters and applied to a HisTalon gravity column (Clontech Laboratories, Inc.), pre‐equilibrated with the same buffer. The column was further washed with buffer R containing 10 mM imidazole. Elution was then performed using buffer R containing 50 mM imidazole. Purification efficiency was evaluated by sodium dodecyl sulfate‐polyacrylamide gel electrophoresis (SDS‐PAGE) (Lee et al., 2009), and the fractions containing the highest concentrations of the target protein were merged. Gel‐filtration on a PD‐10 column (GE Healthcare) was used to remove the excess of imidazole. The purified fractions were then dialyzed against storage buffer (20 mM Tris‐HCl, 40 mM KCl, 0.1 mM DTT, 0.1 mM EDTA, 0.5% Tween‐20, 0.5% Nonidet P40, and 50% glycerol) (Gibbs et al., 2009) and stored at −20°C for further investigation. The final protein concentrations were measured using a Qubit Assay kit (Life Technologies).

### 2.4 Activity assay

Polymerase activity was evaluated using a method described previously (Choi et al., 2006) with minor modifications. Briefly, the standard reaction mixture (50 μL) contained 20 mM Tris‐HCl (pH 7.5); 40 mM KCl; 2 mM MgCl_2_; 100 mM dCTP, dATP, and dGTP each; 10 mM dTTP; 0.5 μCi [^3^H] thymidine 5’‐triphosphate (2.59–3.33 TBq/mmol; PerkinElmer); 1.25 μg activated calf thymus DNA; and 0.5 μL *Ts*K1 DNA polymerase solution. The mixture was incubated at 70°C for 10 min, and the reaction was terminated by placing the mixture on ice and adding 0.5 M EDTA. Subsequently, an aliquot was applied onto a DE81 filter‐paper disc (23 mm; Whatman), which was then dried on a heat block, washed with 0.5 M sodium phosphate (pH 7.0) buffer for 10 min and in 70% (v/v) ethanol for 5 min, and dried. The radioactivity incorporated in the dried filter‐paper disc was then measured using a Tri‐Carb 2900 TR Liquid Scintillation Analyser (PerkinElmer).

To determine the optimal pH, three buffer systems with different buffering capacities (MOPS‐NaOH [pH 6.0–8.0], Tris‐HCl [pH 8.0–9.5], and glycine‐NaOH [pH 9.0–10.0] at 25°C) were tested using this assay system.

To determine the optimal temperature, the reaction mix containing the optimal buffer was incubated at various temperatures (45–80°C) for 10 min and then subjected to the above‐described activity assay.

The influence of K^+^ ions on polymerase activity was determined by adding various concentrations (from 0 to 200 mM) of KCl to the basic reaction mixture and the dependence of the polymerase on divalent cations (Mg^2+^, Mn^2+^) was determined using various concentrations (from 0 to 20 and from 0 to 10 mM, respectively) of MgCl_2_ and MnCl_2_.

Various dilutions of the enzyme solution were used in each reaction under optimal pH and temperature conditions to determine the specific activity of *Ts*K1 DNA polymerase. One unit of *Ts*K1 DNA polymerase was defined as the amount of enzyme needed to convert 10 pmol of [^3^H] TTP into an acid‐insoluble product at 72°C in 10 min.

To investigate the thermostability properties of this enzyme, we subjected the purified enzyme, without any additional stabilizers, to 75, 80, 88, and 95°C for up to 1 h. Aliquots of the enzyme were removed at 5, 15, 30, 45, and 60 min and quenched on ice. The residual activity of these samples was then determined in the optimal reaction mix as described above.

All measurements were carried out in triplicate.

### 2.5 Fidelity assay

The fidelity assays were performed using the blue‐white screening method described previously (Lee et al., 2009) with some modifications. Briefly, primers pUC19_F (5′‐gcatgaAAGCTTGCATGCCTGCAGGTCGAC‐3′) and pUC19_R (5′‐gcatgaCATATGCGGTGTGAAATACCGCAC‐3′), which incorporate a *Hin*dIII and *Nde*I site, respectively (underlined), were used to amplify a 265 bp fragment of the *lac*Zα gene using pUC19 as a template. The primers were designed manually using the Primer3 online tool (https://www.ncbi.nlm.nih.gov/tools/primer‐blast/), and PCR was performed using *Ts*K1 DNA polymerase, OneTaq (NEB), Fusion (Thermo Fisher), and *Taq* (Sigma) DNA polymerases in their optimal buffers and assayed under their optimal reaction conditions. The PCR products were then digested with *Hin*dIII and *Nde*I, purified, and ligated to the 2421 bp *Hin*dIII/*Nde*I fragment from pUC19. Each ligation mixture was then used to transform chemically competent *E*.* coli* TOP10 cells. Transformed cells were plated on LB agar plates supplemented with 100 μg/ml carbenicillin, 40 μg/ml X‐gal, and 0.3 mM IPTG. Pale blue and white colonies were formed by cells containing mutant plasmids, while blue colonies were formed by cells containing wild‐type plasmids. The percentage of white and pale blue colonies was then calculated.

### 2.6 PCR assays

PCRs using *Ts*K1 DNA polymerase were then performed using optimized buffer (10 mM Tris‐HCl (pH 9.0), 50 mM KCl, 0.1% Triton X) under the optimal cycling conditions (extension at 68–72°C for 1 min/kb, annealing for 20–30 s, and denaturation at 94°C for 20–30 s). Various commercially available and manually designed (using Primer3 online tool (https://www.ncbi.nlm.nih.gov/tools/primer‐blast/)) primer sets were applied using both genomic and plasmid DNA as a template (Table 1) to create amplicons of different sizes.

**Table 1:** Primers and templates used for testing the amplification ability of *Ts*K1

Primer	Sequence, 5′−3′	Template	Amplicon size	Reference
K517r^a^	ATTACCGCGGCTGCTGG	Bacterial genomic DNA	500 bp	Muyzer et al., 1993
A8‐28F^a^	AGAGTTTGATCCTGGCTCAG	Bacterial genomic DNA	500 bp	Edwards et al., 1989
27_F^a^	GAGTTTGATCCTGGCTCAG	Bacterial genomic DNA	1.5 kb	Woese et al., 1983
R13^a^	AGAAAGGAGGTGATCCAGCC	Bacterial genomic DNA	1.5 kb	Dorsch & Stackebrandt, 1992
T7^a^	TAATACGACTCACTATAGGG	Plasmid p7xC3H	1929 bp	https://www.addgene.org/mol‐bio‐reference/sequencing‐primers/
T7_term^a^	GCTAGTTATTGCTCAGCGG	Plasmid p7xC3H	1929 bp	https://www.addgene.org/mol‐bio‐reference/sequencing‐primers/
Puc19_f^b^	GCATGAAAGCTTGCATGCCTGCAGGTCGAC	Plasmid pUC19	265 bp	This work
Puc19_r^b^	GCATGACATATGCGGTGTGAAATACCGCAC	Plasmid pUC19	265 bp	This work
Tpol1_F^b^	ATATCATATGCTGCCGCTGTTTGAGCCGAAGG	pUC57‐Mini plasmid harboring polI gene	2.5 kb	This work
Tpol1_R^b^	TATACTCGAGTGCCGCCTTCGCGCTCAGCCAG	pUC57‐Mini plasmid harboring polI gene	2.5 kb	This work

^a^ Commercially available primers,

^b^ Manually designed primers

## 3 RESULTS

### 3.1 Polymerase gene

**FIGURE 1:**
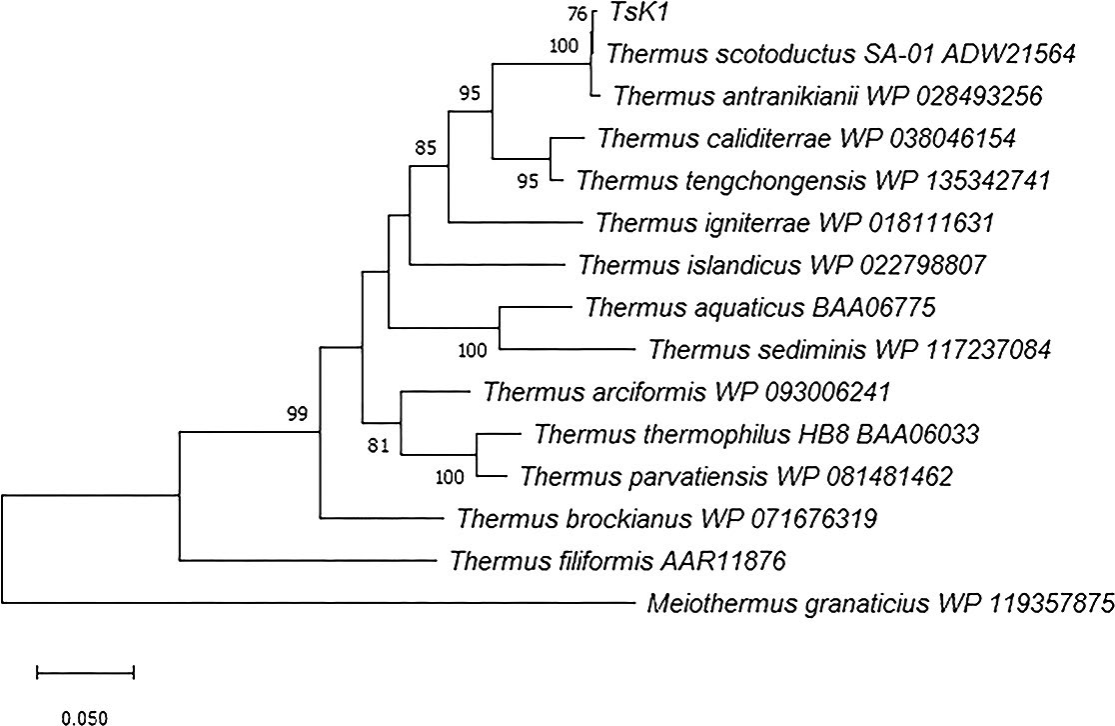
Phylogenetic tree showing the evolutionary relationship between *Ts*K1 DNA polymerase and other *Thermus* DNA polymerases based on maximum likelihood analysis. The tree with the highest likelihood (7699.30) is shown, and the bootstrap value (1000 replicates) for each clade is shown next to each branch. The final dataset contained 824 positions. All positions containing gaps or missing data were eliminated (complete deletion option). Bar indicates 0.05 substitutions per amino acid position. Evolutionary analyses were conducted using MEGA X

The original *Ts*K1 DNA polymerase gene encompassed 2496 bp and encoded an 830 amino acid protein with an estimated molecular mass of 93,613 Da. The alignment of several related amino acid sequences from various *Thermus* species, available in the NCBI database was used to perform phylogenetic analysis. The phylogenetic tree, rooted using *Meiothermus granaticus* (Figure 1), showed that *Ts*K1 DNA polymerase was very closely related to the polymerases from *T*.* scotoductus* SA‐01 and *T*.* antranikianii* and distinct from the DNA polymerase I enzymes from *T*.* aquaticus* and *T*.* thermophilus*. Moreover, it shared 85.75% sequence similarity with native *Taq* polymerase.

### 3.2 Expression and purification of *Ts*K1 DNA polymerase

**FIGURE 2:**
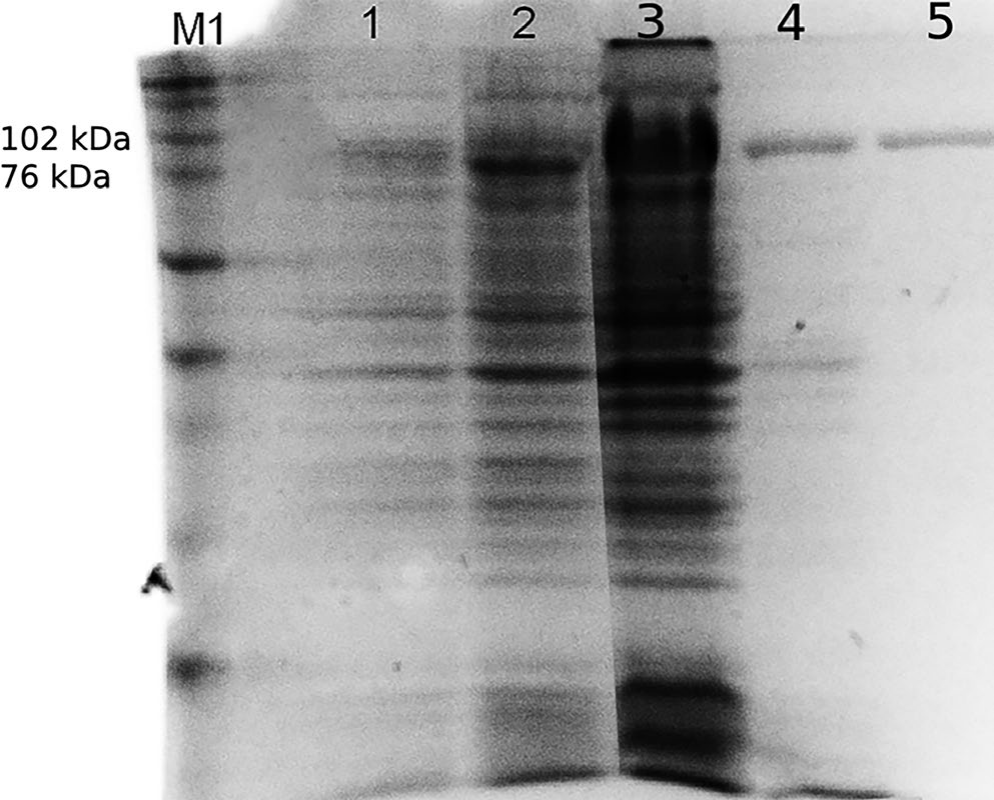
Sodium dodecyl sulfate‐polyacrylamide gel electrophoresis of *Ts*K1 DNA polymerase purification: (1) noninduced culture, (2) induced culture, (3) sonicated extract from host cells, (4) supernatant after heat treatment, 5) purified protein, M1—Full Range Rainbow molecular‐mass marker (Amersham)

The overexpression of the desired protein in *E*.* coli* was achieved successfully. SDS‐PAGE analysis indicated that the recombinant protein was approximately 94 kDa (Figure 2, lanes 1 and 2), which agrees with the molecular mass calculated based on amino acid sequence, supporting our conclusion that the correct full‐length protein was expressed in this system. In the sonicated extracts, the presence of a significant amount of 94 kDa protein (Figure 2, lane 3) suggests that the expressed protein is soluble. Reduced bands and background following heat treatment suggests successful precipitation of most host proteins (Figure 2, lane 4), and the high degree of homogeneity following affinity chromatography (Figure 2, lane 5) confirmed the presence of a highly purified protein.

### 3.3 Optimal conditions for *Ts*K1 DNA polymerase activity

**FIGURE 3:**
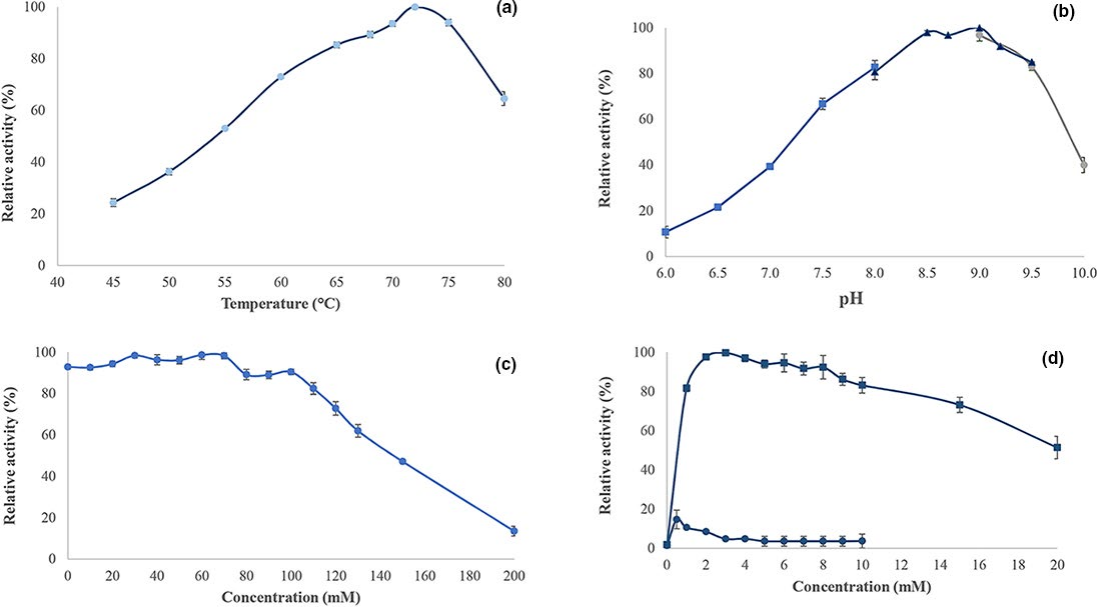
Properties of *Ts*K1 DNA polymerase. (a) Effect of temperature on *Ts*K1 DNA polymerase activity; (b) effect of pH on *Ts*K1 DNA polymerase activity in MOPS‐NaOH (■), Tris‐HCl (▲), and glycine‐NaOH (●) buffers; (c) effect of KCl concentration on *Ts*K1 DNA polymerase activity; and (d) effect of the divalent cations Mg^2+^ (■) and Mn^2+^ (●) on *Ts*K1 DNA polymerase activity. Each point represents the average of 3 measured values, and error bars represent the standard deviation between these 3 values

We evaluated *Ts*K1 DNA polymerase activity between 45 and 80°C and determined that the optimal temperature range for this enzyme was 68–75°C, and the highest activity was observed at 72°C (Figure 3a). An evaluation of the pH requirements for this enzyme revealed that it was most active between pH 8.5 and 9.2 in Tris‐HCl buffer, demonstrating the highest activity at pH 9.0 (Figure 3b). To study the effects of divalent cations on *Ts*K1 DNA polymerase activity, we evaluated various concentrations of MgCl_2_ and MnCl_2_ and confirmed that the enzyme was highly dependent on Mg^2+^ ions. We identified that the Mg^2+^ ion concentration of 3–5 mM was optimal for the activity of this enzyme, whereas a concentration of >8 mM led to decreased activity. The presence of Mn^2+^ ions had almost no influence on polymerase activity (Figure 3d).

**FIGURE 4:**
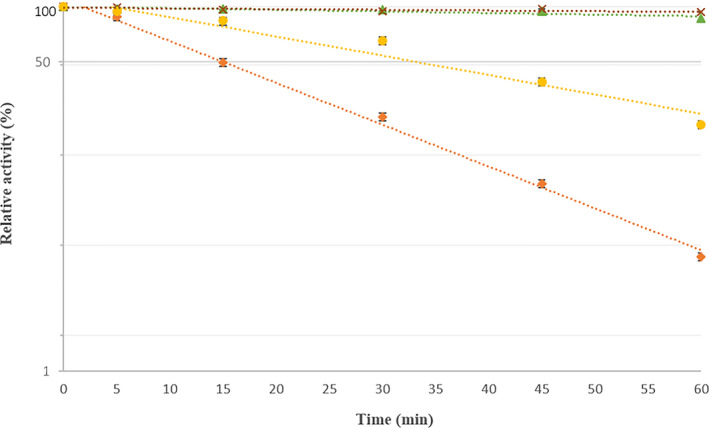
*Ts*K1 DNA polymerase thermostability. ×Represents 75°C, ▲ represents 80°C, ● represents 88°C, ♦ represents 95°C. Data are represented on a logarithmic scale. Each point represents the average of 3 measured values, and error bars represent the standard deviation between these 3 values

The influence of KCl on *Ts*K1 DNA polymerase activity is shown in Figure 2c and demonstrates that this enzyme was active at up to 70 mM KCl and that higher concentrations of potassium reduced the enzyme activity of *Ts*K1 DNA polymerase. The specific activity of pure *Ts*K1 DNA polymerase was calculated to be 27 units/µg. We went on to examine the thermostability of *Ts*K1 DNA polymerase and determined that the enzyme was stable at temperatures of 75 and 80°C and was relatively stable at 88°C. It lost half of its activity after 15 min of incubation at 95°C (Figure 4).

### 3.4 *Ts*K1 DNA polymerase fidelity

More than 33,000 colonies were counted from the *Ts*K1 ligation mix, and approximately 5000 colonies were counted for each of the other enzymes. The proportion of mutant (white) colonies were calculated to be 1.26%, 2.40%, 2.39%, and 0.7792% for *Ts*K1, *Taq*, OneTaq, and Fusion polymerase, respectively. Various mutant colonies were picked from each experiment and tested using the same primer pair used in the initial amplification. Besides colonies harboring amplicons with the correct size (265 bp), several tested colonies containing ‘double inserts’ were detected as well.

### 3.5 PCR by *Ts*K1 DNA polymerase

**FIGURE 5:**
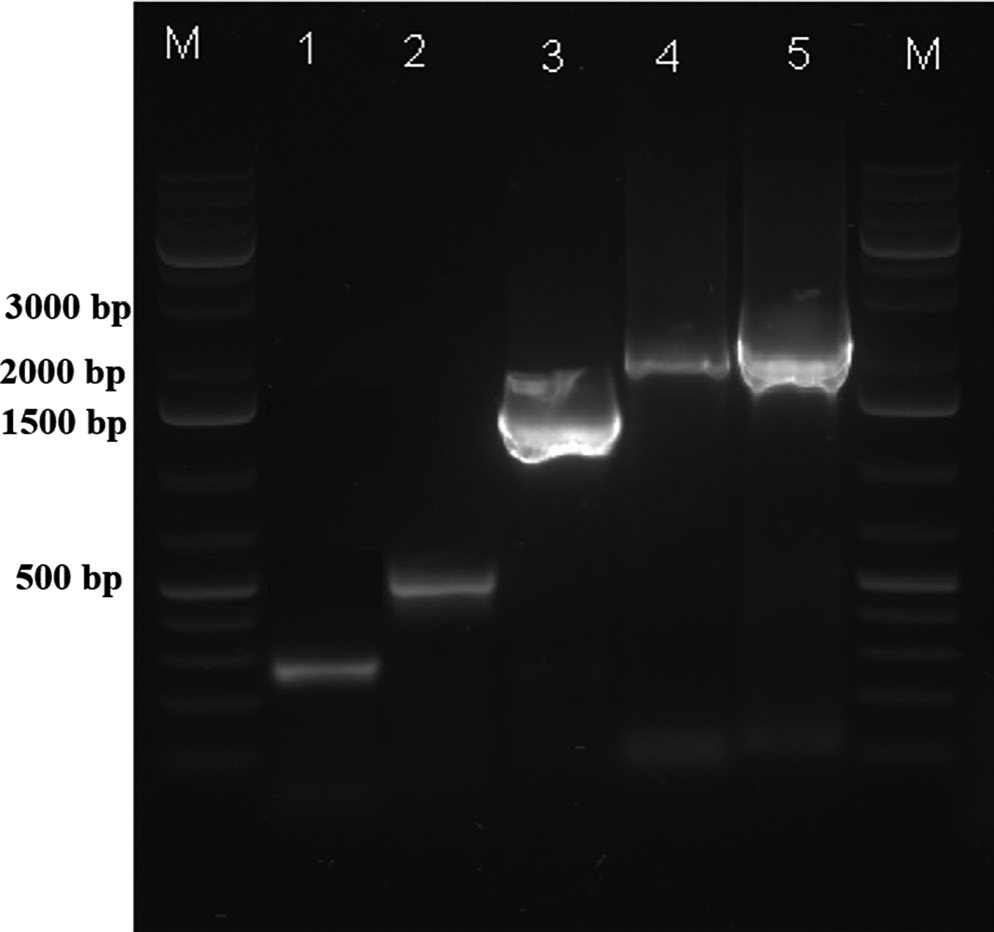
Polymerase chain reaction products amplified using *Ts*K1 DNA polymerase. Lane 1, 265 bp; lane 2, 500 bp; lane 3, 1500 bp; lane 4, 1920 bp; lane 5, 2.5 kb; M, GeneRuler 1 kb Plus DNA ladder (New England BioLabs)

The electrophoretogram of the PCR products amplified by using the *Ts*K1 DNA polymerase is shown in Figure 5. Lanes 2 (500 bp) and 3 (1.5 kb) represent amplicons from bacterial genomic DNA, and lanes 1 (265 bp), 4 (1920 bp), and 5 (2.5 kb) represent products amplified from plasmid templates. The high intensities of the bands suggest that *Ts*K1 DNA polymerase can amplify products of up to 2.5 kb with high efficiency.

## 4 DISCUSSION

The expression, purification, and characterization of a DNA polymerase I from *T*.* scotoductus* K1 are described in the current study. The amino acid sequence comparison showed that this enzyme shares a high degree of similarity to DNA polymerase from *T*.* scotoductus* SA‐01. This finding was expected, as according to earlier evaluations of the 16S rRNA gene sequences from these bacteria, they showed >99% similarity (Saghatelyan et al., 2015). *Ts*K1 DNA polymerase is relatively different from *T*.* aquaticus* DNA polymerase, which might explain the differences in the behavior of this enzyme as discussed below.

The FX cloning strategy used to clone *Ts*K1 DNA polymerase was based on the use of type IIS restriction enzymes, which digest DNA at an exact distance from their asymmetric recognition sites. The resulting overhang is defined only based on its distance from the recognition site and not by its sequence. Following digestion, the recognition sites are physically separated from the cleavage site, minimizing cloning‐related artifacts in the open reading frame (Geertsma & Dutzler, 2011). The optimization of coding triplets facilitated the proper expression of this thermophilic enzyme in a mesophilic host, thus yielding a significant amount of soluble enzyme. Although heat treatment and further centrifugation eliminated most host proteins, some undesired proteins were still noted in the supernatant (Figure 2, lane 4). The HisTalon gravity column is a cobalt‐charged tetradentate chelator, specific for his‐tagged proteins, and has rigorous requirements for the 3D positioning of histidine residues (only contiguous histidine residues or specially arranged, surrounding histidine residues can bind the cobalt in the reactive core) compared with most commonly used nickel‐based resins. This allows more specific binding of the recombinant His‐tagged *Ts*K1 DNA polymerase, resulting in an almost pure protein (Figure 2, lane 5).

Following optimization of buffering conditions, *Ts*K1 DNA polymerase demonstrated the highest activity at pH 9.0 in Tris‐HCl buffer, which is different from that of *Taq* that prefers a less alkaline pH (Chien et al., 1976; Lawyer et al., 1993). Polymerase I from *T*.* caldophilus* GK24 requires an optimum pH of 8.7 (Park et al., 1993).

*Ts*K1 DNA polymerase activity was rather independent of KCl at lower concentrations, although high concentrations of this salt did inhibit its enzymatic activity. Reportedly, KCl at concentrations above 100 mM inhibits *Taq* polymerase as well (Chien et al., 1976). Although *Ts*K1 DNA polymerase does not appear to be dependent on KCl when using calf thymus DNA as a template, the influence of KCl may differ with different enzymes and different templates (Lawyer et al., 1993

).

Divalent cations are necessary for polymerization. Here, we demonstrated that the *Ts*K1 DNA polymerase activity was dependent on Mg^2+^ to some extent. This is in agreement with the literature, which suggests that Mg^2+^ is a critical component for polymerases (Choi et al., 2006). For *Ts*K1 DNA polymerase, the optimal concentration of Mg^2+^ is 3–5 mM, in contrast to that for *Taq* (10 mM), recombinant *Taq* (2–4 mM), and *Tca* (12 mM) (Chien et al., 1976; Lawyer et al., 1993; Park et al., 1993).

The optimal temperature for *Ts*K1 DNA polymerase was shown to be 72–74°C, which is comparable with other Taq‐like polymerases (Lawyer et al., 1993; Park et al., 1993) and the lower polymerization activity described at 80°C could also be explained by the template (activated calf thymus DNA) denaturation at higher temperatures, as the *Ts*K1 DNA polymerase was stable at 80°C.

Although the half‐life of *Ts*K1 DNA polymerase (without the use of additional stabilizers) at 95°C (15 min) is lower than that of *Taq* (Lawyer et al., 1993), it is close to another commercially available r*Taq* (20 min) polymerase (Gibbs et al., 2009) suggesting that it could still be used in PCR based applications. Furthermore, this characteristic could be enhanced through the application of directed mutagenesis techniques. Some polymerases from *Thermus* strains show lower thermostability than *Ts*K1 DNA polymerase; these include *Wai28A.1, NMX2A.1, Fiji3A.1, and OHA.2* with a half‐life of 4, 3.5, 6, and 2.5 min, respectively, at 95°C. However, these enzymes also show reduced PCR amplification efficiency compared with *Taq* (Gibbs et al., 2009). In contrast, *Ts*K1 DNA polymerase demonstrated high amplification efficiency using various templates (Figure 5). Of the other *Thermus* polymerases, only *Tca* polymerase has been shown to exhibit high thermostability (70 min at 95°C in the presence of gelatin) (Park et al., 1993).

Numerous dilations to the basic PCR approach have been reported and respective enzymes with various degrees of similarity to *Taq* polymerase have been mined from other *Thermus* spp. (Gibbs et al., 2009). Gibbs and colleagues compared six recombinant polymerases originating from different *Thermus* species, which were selected based on their degree of divergence, and demonstrated that all of these enzymes retained similar biochemical characteristics. Moreover, some studies suggest that the other polymerases originating from *Thermus* spp. can be more efficient in certain cases of PCR in contrast to *Taq*. For instance, it has been reported, that the reverse transcriptase activity of a recombinant DNA polymerase from *T*.* thermophilus* was 100‐fold higher than the RT activity of *T*.* aquaticus* DNA *pol*, although these two strains are closely related (Gibbs et al., 2009). These and other results indicate that few or even one residue variations of polymerases may have a dramatic influence on their biochemical specifications.

PCR itself is a complicated reaction and depends on several factors including polymerase activity, template type, and the concentration of other components. KCl facilitates the elongation of the primer‐DNA complex, and the activity of thermostable DNA polymerases is promoted by K^+^ ions which guard the negatively charged DNA backbone. This binding decreases the electrostatic repulsion between the two DNA strands and facilitates preferential elongation. During PCR, K^+^ ions have an optimum promoting effect on DNA polymerases at a concentration of about 50 mM (van Pelt‐Verkuil et al., 2008). Thus, a *Ts*K1 DNA polymerase buffer containing 50 mM KCl and 3 mM MgCl_2_ was shown to be optimal for the amplification of 2.5 kb products using various templates (Figure 5).

Our results suggest that *Ts*K1 DNA polymerase is twice as accurate as *Taq* but approximately 1.6 times less accurate than Fusion polymerase. However, Fusion polymerase is approximately 52 times more accurate than *Taq* (http://tools.thermofisher.com/content/sfs/brochures/phusion‐dna‐polymerase‐brochure.pdf). These results can be explained by the limitations of the method used. First, the fragment size amplified by these enzymes was relatively small (265 bp), thus reducing the chance of an incorrect inclusion. Next, the examination of a few mutant colonies indicated the presence of so‐called double inserts, which resulted in defective *lacZ*, but these inserts may not be mutated. Also, several ‘silent’ mutations may occur and produce active *lacZ* but still harbor a mutation. However, some studies using similar methods for fidelity testing indicated mutation frequencies of 2.3, 1.3, 0.85, 3, and 2.3% for r*Taq, OHA.2, Wai28A.1, NMX2A.1*, and *Fiji3A.1* polymerases, respectively (Gibbs et al., 2009). Even with these limitations, we can still confidently assert that *Ts*K1 DNA polymerase is at least 2 times more accurate than *Taq* but further investigations are needed to confirm this finding.

## 5 CONCLUSION

Although *Taq* is the most commonly used polymerase enzyme, there are several other enzymes from other strains of *Thermus* that exhibit properties similar to *Taq* polymerase. Here, we describe a novel DNA polymerase from the newly isolated extreme thermophilic *T*.* scotoductus* K1. *Ts*K1 DNA polymerase was cloned and expressed using an *E*.* coli* host, then purified and characterized. Optimization of the expression and purification conditions yielded a high concentration of recombinant *Ts*K1 DNA polymerase, and this enzyme was shown to exhibit high PCR efficiency. This suggests that *Ts*K1 DNA polymerase may be a good candidate for the development of novel molecular biology methodologies.

## CONFLICT OF INTEREST

None declared.

## AUTHOR CONTRIBUTION

**Ani Saghatelyan:** Conceptualization (equal); Formal analysis (equal); Visualization (lead); Writing‐original draft (lead); Writing‐review & editing (equal). **Hovik Panosyan:** Conceptualization (equal); Formal analysis (equal); Funding acquisition (equal); Project administration (equal); Supervision (equal); Writing‐review & editing (equal). **Armen Trchounian:** Writing‐review & editing (supporting). **Nils‐Kaare Birkeland:** Conceptualization (equal); Formal analysis (equal); Funding acquisition (equal); Project administration (equal); Supervision (equal); Writing‐review & editing (lead).

## ETHICS STATEMENT

None required.

## Data Availability

The nucleotide sequence of the synthetic codon‐optimized *polI* gene encoding the *Ts*K1DNA polymerase is available in GenBank under accession number MW080815: https://www.ncbi.nlm.nih.gov/nuccore/MW080815
